# Different Preparation Method of Nanocellulose from *Macaranga gigantea* and Its Preliminary Study on Packaging Film Potential

**DOI:** 10.3390/polym14214591

**Published:** 2022-10-28

**Authors:** Latifah Jasmani, Nurul Ain Nadirah Jamaluddin, Rafeadah Rusli, Sharmiza Adnan, Sarani Zakaria

**Affiliations:** 1 Forest Products Division, Forest Research Institute Malaysia (FRIM), Kepong 52109, Selangor, Malaysia; 2 Faculty of Science and Technology, Universiti Kebangsaan Malaysia (UKM), Bangi 43600, Selangor, Malaysia

**Keywords:** cellulose, Macaranga, packaging, crystalline, fibril

## Abstract

Nanocellulose is a versatile cellulosic nanomaterial that can be used in many application areas. Applying different preparation strategies leads to different types of nanocellulose. In this study, nanocrystalline cellulose (NCC) and nanofibrillated cellulose (NFC) were prepared from lesser known wood species, viz., *Macaranga gigantea*, using sulfuric acid hydrolysis and enzymatic pretreatment with ultrafine grinding approaches, respectively. The respective nanocellulose was characterized by means of Fourier transform infrared spectroscopy (FTIR), X-ray diffraction (XRD) analysis, thermogravimetric analysis (TGA), atomic force microscopy (AFM). It was then converted into a thin film to assess its performance which includes tensile test, transparency, air permeance, water vapor transmission rate (WVTR), and water vapor permeability (WVP) properties. NCC and NFC produced from the raw material of Macaranga had average widths of 6.38 ± 3.92 nm and 13.17 ± 12.71 nm, respectively. Peaks in FTIR spectra showed the conversion of Macaranga wood to nanocellulose by the presence of cellulose fingerprint as well as absence of lignin and hemicellulose after alkaline treatment. The successful conversion was also supported by XRD analysis which displayed the increased crystallinity value from 54% to 70%. TGA decomposition pattern at 200–490 °C revealed the thermal stability of the samples. The thin film produced from nanocelluloses had WVTR values of 4.58 and 12.14 g/(day·m^2^) for NFC and NCC, respectively, comparable to those of films from polyester and oriented polypropylene. Nanocellulose-based thin film has the potential to be used as sustainable and biodegradable packaging.

## 1. Introduction

Nanocellulose has been actively studied over the past two decades. Its popularity is still growing as a result of its sustainability, biodegradability, and renewable nature, as well as the global need for biobased and sustainable products for use in a variety of applications. Not only does it offer alternative use to nonsustainable materials, nanocellulose also possesses remarkable attributes by having a high surface area, high mechanical strength, distinct optical properties, and low toxicity [1,2,3].

Nanocellulose derived from plant material is basically categorized into two types, namely, nanocrystalline cellulose (NCC) and nanofibrillated cellulose (NFC). NCC is typically prepared from acid hydrolysis, with sulfuric acid being the most widely used [4,5]. Other types of acids, such as hydrochloric acid [6] and hydrobromic acid [7], have also been reported. Sulfuric acid has gained popularity for NCC preparation over other acids due to the side reaction that takes place, i.e., esterification, which leads to the formation of sulfate groups on the surface of NCC. The presence of these negatively charged groups leads to electrostatic repulsion that makes the suspension of NCC more stable. In addition to acid hydrolysis, work on the use of ionic liquid to extract NCC has also been published by some researchers [8,9]. While the majority of NCC extraction work involves the use of acid and a series of purification processes to remove residual acid, NFC preparation, on the other hand, offers a much simpler approach and is usually achieved via mechanical treatment. Unlike NCC, NFC can be prepared using different machines, such as grinders, homogenizers or microfluidizers. The properties of nanocellulose vary with the reaction conditions and source of cellulose [10].

Owing to its unique optical and physicochemical characteristics as well as high mechanical properties, nanocellulose can be applied across different industrial needs, such as advanced composites [11], paper packaging [12,13,14], cosmetics, medicine [15], energy storage [16], and many others. One of the areas that has gained increasing attention in view of green products is paper-based/cellulose-based packaging. The global drive for sustainable or environmentally friendly packaging is due to landfill and environmental issues caused by non-biodegradable petroleum-based plastic, in which tonnes of plastic have been dumped in the landfill every day across the globe. Numerous efforts have been made to produce biodegradable plastics such as polylactic acid (PLA) [17] or polyhydroaxyalkanoate (PHA). Nevertheless, the production of such bioplastics (especially PLA) typically competes with food sources, as they are mostly made from sugar extracted from corn, sugarcane, or tapioca. As nanocellulose can be produced from various lignocellulosic materials and can also be converted to thin film/nanopaper or plastic-like materials, it can become another alternative for use in packaging. Although much work has been conducted on the utilization of nanocellulose in coatings, films, and layers for packaging [18], very little information is available on the use of tropical hardwood for pure nanocellulose-based thin films for packaging potential. In addition, the conversion of lesser known wood species, i.e., *Macaranga gigantea* to high value intermediate products such as nanocellulose could lead to the production of a plethora of high value and sustainable end products. In this study, NCC and NFC are prepared from *Macaranga gigantea* via acid hydrolysis and a combination of enzymatic pretreatment and grinding techniques, respectively, followed by corresponding characterization. The NCC and NFC in the form of thin films were also evaluated for their potential use in packaging applications as alternatives to plastic. However, in this study, the thin film from the two types of nanocellulose was only evaluated for its tensile strength, transparency, air permeance, WVTR, and WVP properties.

## 2. Materials and Methods

### 2.1. Chemicals

Cellulase enzyme (*Trichoderma reesei* (ATCC 26921)) was purchased from Sigma Aldrich (St Louis, MO, USA). Sodium sulfide (>60%) (Na_2_S), sodium hydroxide (min 98%) (NaOH), ethanol (95%) (C_2_H_5_OH), anhydrous calcium chloride (>74%) (CaCl_2_), hydrofluoric acid (55%) (HF), hydrochloric acid (37%) (HCl), and toluene (min 99%) (C_7_H_8_) were obtained from Chemiz (Shah Alam, Malaysia), whereas glacial acetic acid (99.8%) (CH_3_COOH) was obtained from Ajax Chemicals (Melbourne, Australia), and sodium chlorite (>79%) (NaClO) was obtained from JT Baker (Radnor, PA, USA). Sulfuric acid (96%) (H_2_SO_4_) was obtained from Merck (Darmstadt, Germany).

### 2.2. Raw Material Preparation

*The Macaranga gigantea* used in this study is readily available on the FRIM campus as a pioneer species. The tree was chopped, cut into logs and subsequently processed into wood chips using a wood chipper. The wood chips of selected dimensions were then cooked via kraft pulping in a rotary digester at 170 °C for 3½ h. After pulping, the pulp was bleached to remove the residual lignin to obtain lignin-free pulp. The bleaching process was conducted via a 5-stage DEDED sequence in which D is denoted for chlorine dioxide as the bleaching agent and E is for the extraction stage, which involved the use of sodium hydroxide. The bleached pulp was used as feedstock for the preparation of NFC and NCC.

### 2.3. Fibre Morphology

Wood fibers were produced by digestion of wood splints in a water bath with consecutive additions of acetic acid and sodium chlorite. In a bottle, softened wood splints were shaken with glass beads. The fibers were placed on a glass slide and dyed with safranin-o to enhance their visibility. The length, width, and wall thickness of the fibers were measured using a stereomicroscope.

### 2.4. Chemical Composition

A wood disc was cut from a *Macaranga gigantea* tree at breast height. The disc was debarked, sliced into matchstick sizes, and finely ground by a Wiley mill. The ground samples were screened through a 40 mesh screen, and samples retained on a 60 mesh filter were used for chemical analysis. Afterwards, the samples were air dried for at least one day. The chemical analysis was carried out in accordance with the standard protocols by TAPPI and other methods as follows: hot water soluble (TAPPI 207 cm-99), 1% alkali soluble (TAPPI 212 om-02), extractives (TAPPI 204 cm-97), ash content (TAPPI 211 om-02), alpha-cellulose content (TAPPI 203 cm-99), lignin content (TAPPI 222 om-02), pentosans [19], and holocellulose content [20].

### 2.5. NFC Preparation

The bleached pulp was pretreated with cellulase enzyme to facilitate the subsequent defibrillation stage. The enzyme-treated pulp was introduced to ultrafine grinding using Masuko Sangyo Grinder (MKCA6-2) for multiple cycles until gel-like NFC was formed.

### 2.6. NCC Preparation

To the bleached pulp in a beaker, 400 mL sulfuric acid (64%) was added. The hydrolysis was conducted for 40 min at 45 °C, and the mixture was stirred. After 45 min, cold water was added to stop the hydrolysis. The resulting NCC was centrifuged to remove the acid and dialyzed against tap water until the pH suspension in the tubing was neutral.

### 2.7. Fabrication of Thin Film from Nanocellulose

The thin film was produced from the respective suspension of nanocellulose at 0.5 wt%. The suspension was sonicated using a tip sonicator (QSonica) for 2 min to ensure that it was free from aggregates. The suspension was cast on a Petri dish and dried in an oven at 50 °C. The thin film was peeled off from the Petri dish and subjected to tensile test, transparency, air permeance, WVTR and WVP analysis.

**Scheme 1:**
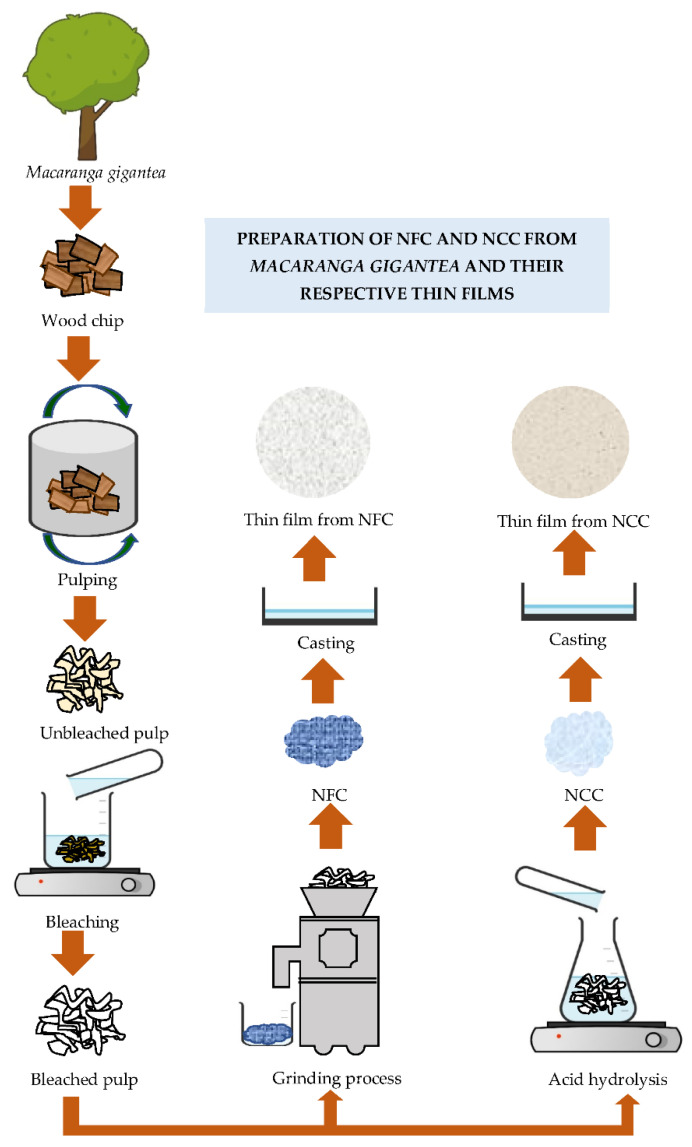
Schematic diagram for the preparation of NFC and NCC from raw material i.e., *Macaranga gigantea* and their corresponding thin films.

Scheme 1 represents a scheme of the preparation of NFC and NCC from raw Macaranga and also the thin films produced from the respective nanocellulose.

### 2.8. Characterization

#### 2.8.1. Atomic Force Microscopy (AFM)

Samples of nanocellulose were prepared in a 0.01% *w/w* suspension and dropped on freshly cleaved mica sheets. The mica was dried at room temperature and subjected to imaging via an atomic force microscope (Bruker Innova, Shah Alam, Malaysia) in tapping mode.

#### 2.8.2. Fourier Transform Infrared Spectroscopy (FTIR)

Samples were homogenized with potassium bromide (KBr) and ground into a powder. The mixture was transferred into a mold and pressed using a hydraulic press to form a KBr disc. FTIR spectra were recorded using a Perkin Elmer Spectrum 100 series in the range of 4000 to 450 cm^−1^ with a total accumulation of 16 scans.

#### 2.8.3. X-ray Diffraction (XRD)

Powder X-ray diffraction (XRD) spectra were acquired using PANalytical X’Pert Pro MD in Bragg-Brentano geometry, with monochromated CuK_α1_ radiation (λ = 1.5406 Å), automated divergence and receiving slits (10 mm illuminated length), 10 mm beam mask, 0.04 rad soller slits, and a step size of 0.08°. The nanocellulose was placed on a zero background silicon plate, rotated in the X-ray chamber, and investigated under X-ray radiation generated at 40 mA and 40 kV.

The crystalline indices of the samples can be calculated using the following equation.

(1) $$\mathrm{Crystalline}\;\mathrm{index}=\frac{I_{200}-I_{am}}{I_{200}}$$

**Equation (1)**. Crystalline index, where *I*_200_ is the peak from the 200 lattice plane (2θ = 22.5°) and *I_am_* is the peak intensity of the amorphous phase (2θ = 18°).

#### 2.8.4. Thermogravimetric Analysis (TGA)

Thermogravimetric analysis of all the samples was carried out using a TA Instrument (SDT Q600). The samples in powder form were heated from 30 to 600 °C (10 °C/min) under ambient nitrogen (20 mL/min).

#### 2.8.5. Zeta Potential Measurement

The measurement was carried out using a Malvern Zetasizer ZS. The sample in the form of a suspension (0.1 wt%) was sonicated for 5 min. The zeta potential measurement was made based on electrophoretic light scattering, and Smoluchowski was selected as a model.

#### 2.8.6. Tensile Strength

The thin film samples from the respective nanocellulose were conditioned at 23 °C and 50% relative humidity. The thin films were evaluated for their tensile strength in accordance to MS ISO 1924-2:2010 using Büchel Tensile Tester.

#### 2.8.7. Transmittance

Transparency of both films was determined by the measurement of light transmittance using Jenway UV-Vis Spectrophotometer in the range of 200–800 nm.

#### 2.8.8. Air Permeance, WVTR and WVP Evaluation

The air permeance test was carried out according to the method in ISO 5636-3:2013. The thin sample was placed between a circular gasket and flat surface via Bendtsen Smoothness & Porosity tester and the air flow reading was recorded. The WVTR test was conducted using a Vapometer EZCups (Thwing Albert) in accordance with the TAPPI method (TAPPI T448 om-04). Prior to the test, the weight of the cup, calcium chloride, and thin films was recorded. The calcium chloride was placed inside the cup, and the thin film was sealed to the top part of the cup. The cup was then screw-capped using its O-ring lid. The weight of the thin film was recorded every 24-h interval for 7 days, and the WVTR was calculated according to the method in TAPPI T448 om-04. WVP was calculated from the WVTR value.

## 3. Results and Discussion

*Macaranga gigantea* was selected in this study due to it being a less known wood species but abundantly and easily available in the forest area. As a pioneer species, Macaranga is commonly regarded as species that first appear or easily grow in highly degraded sites or forests. The conversion of Macaranga to cellulosic nanomaterials could add value to the timber itself through product diversification. The chemical composition of Macaranga, as shown in Table 1, can help establish its usefulness for pulp conversion, as it gives an understanding of the quantities of chemical composition of raw material for nanocellulose feedstock. In this study, the values observed for major wood chemical components, namely, holocellulose and alpha-cellulose, are slightly above the average range of Malaysian hardwoods, in which the range reported was between 59.4 to 85.4% and 35.1 to 54.2%, respectively [21]. The cellulose content is related to the expected yield of the chemical pulp. The lignin content of Macaranga, however, is higher than that of most Malaysian hardwood, in which the range reported was 12.7 to 34.2% [21]. The rather high lignin content indicates the tendency to produce pulp with a high Kappa number, which requires more chemicals for the bleaching phase (Haygreen and Boyer 1996). The Kappa number is an indication of residual lignin present in the pulp. The low extractive content for Macaranga is preferable, as it would not affect the end product in terms of odor and appearance.

**Figure 1:**
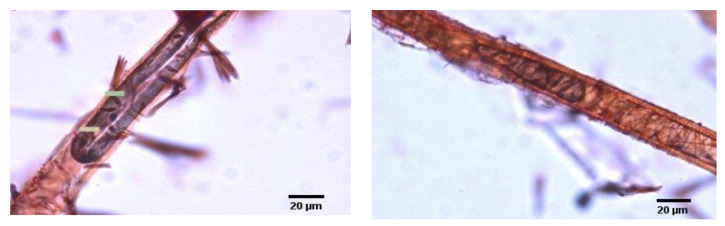
Image of Macaranga fiber under a microscope.

Looking into its fiber dimension, particularly the fiber length, Macaranga is considered a short-fibred material, as shown in Table 2. Fiber length is related to pulp strength, in which the fiber length of Macaranga (1.23 mm) was longer than that of commercially important hardwood species such as *Acacia mangium* (0.98 mm) [22]. The value was also in the range of hardwood fiber viz 0.7 to 1.6 mm [23]. The Runkel ratio should be less than one (1) for any raw material to be used for pulp and paper applications. The Runkel ratio obtained for Macaranga indicates its potential for pulp material, as it would likely form paper of high strength. The fiber is more flexible and would be easily collapsed during papermaking, thus creating more interfibre bonding. The fiber dimension of Macaranga can be clearly observed in Figure 1.

**Figure 2:**
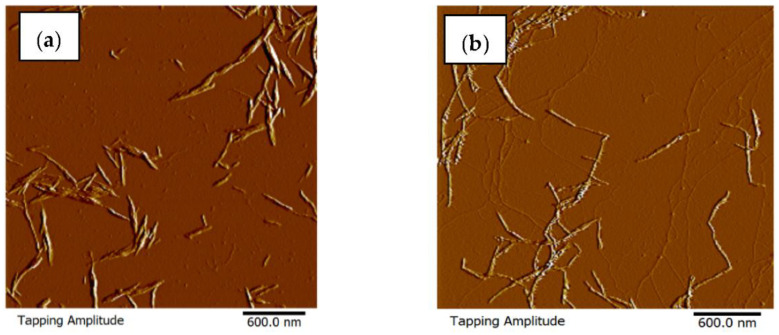
Images of NCC (**a**) and NFC (**b**) observed under AFM.

The preparation of feedstock for nanocellulose in this study covers from primary processing of the wood until the bleaching stage. Primary processing in this study refers to tree cutting, log slicing, and chipping. Upon primary processing of Macaranga timber, the processed wood in the form of wood chips was subjected to chemical pulping to produce lignin-free feedstock for nanocellulose preparation. In this study, kraft pulping was selected for the pulping method to produce a pulp yield of 44.5% and a Kappa number of 16.4. After pulping, the fiber length was slightly affected. The image in Figure 1 shows the liberated fiber after pulping. The fiber bundles were almost absent after chemical digestion, indicating pulping efficiency. The bleached pulp was prepared through a series of bleaching sequences with the objective of removing the residual lignin present in the pulp after the pulping process. As shown in Table 1, the fibers experience some reduction in terms of length and diameter. The reaction during bleaching resulted in minor morphological changes in the fiber. Bleached pulp was subsequently used as a feedstock for nanocellulose preparation. NCC was prepared via the sulfuric acid hydrolysis route, whereas NFC was produced via a combination of enzymatic pretreatment and ultrafine grinding. NCC shown in Figure 2 appeared as a whisker-like structure with an average width and length of 6.38 ± 3.92 nm and 392.60 ± 32.09 nm, respectively (Table 3). The acid diffuses into the lower density of amorphous regions and hydrolyzes them, thus leaving only the tightly packed crystalline domains. The value was close to the width range of NNC prepared from hardwood pulp [24].

Converting bleached pulp into NFC is much less complicated than NCC preparation, as it only involves pretreatment and mechanical disintegration processes. In this study, enzymatic pretreatment was conducted on pulp to facilitate the subsequent fibrillation process via ultrafine grinding. The use of enzymes, particularly cellulase, helps with fibrillation via three possible mechanisms: first, endoglucanase attacks the amorphous domain; second, exoglucanase cleaves the cellulose chain end to produce disaccharides and tetrasaccharides; and cellobiase hydrolyses disaccharides and tetrasaccharides into glucose [25]. During grinding, a slurry of bleached pulp at 2% consistency was passed through rotary and static grinding stones to control the distance between disks. A number of passes is required to reduce the size of the fibers. Delamination of fibers occurs due to shear forces created between the disks. The grinding process was conducted until a gel-like product was formed. The resulting NFC has a width of 13.17 ± 12.71 nm (Table 3). The fibril showed a spaghetti-like or web-like structure.

**Figure 3:**
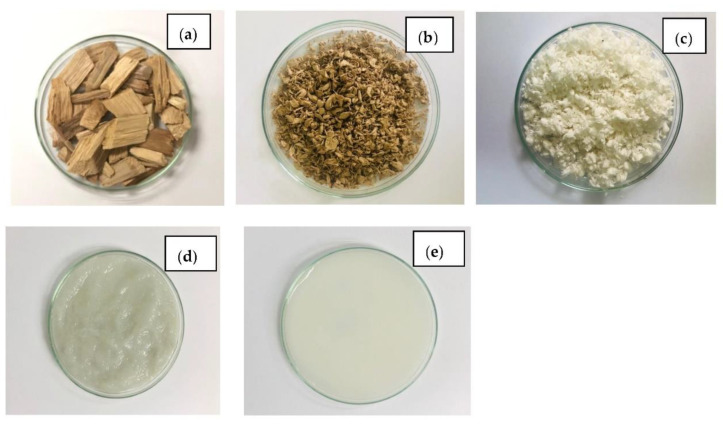
Conversion of *Macaranga gigantea* to NCC and NFC illustrating each step involving (**a**) wood chips, (**b**) unbleached pulp, (**c**) bleached pulp, (**d**) NFC, and (**e**) NCC.

The conversion of Mahang gajah to NFC and NFC is illustrated in each processing stage, as shown in Figure 3. Based on the picture, the striking color difference is before and after pulp bleaching, which is due to the removal of residual lignin in unbleached pulp. After hydrolysis and ultrafine grinding, NCC appeared as an off-white cellulose slurry product (1.8 wt%), whereas NFC is available in a gel-like suspension (4.2 wt%). The suspension of NCC was more stable than NFC due to the electrostatic repulsion created by the formation of sulfate groups during acid hydrolysis. This is supported by the value recorded by zeta potential, in which NCC showed a value of −36.6 mV, whereas NFC showed a value of −31.8 mV. The higher the zeta potential value is, the more stable the system becomes. The charged particles repel each other, and this force overrides the natural inclination to aggregate.

**Figure 4:**
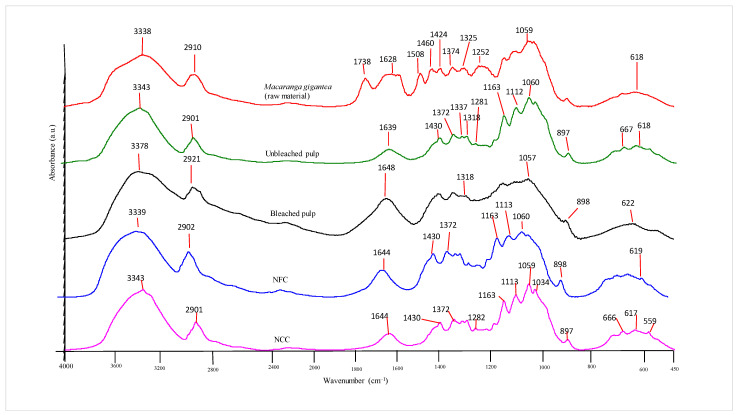
FTIR spectra from each processing stage, i.e., raw material Macaranga to nanocellulose.

To further analyze the functional groups present on the surface of different types of fibers, FTIR analysis was conducted on all samples. The raw material i.e., Macaranga wood shows a distinct band at 1508 cm^−1,^ which corresponds to asymmetric aryl ring stretching present in the lignin compound (Figure 4). The distinct peak at 1738 cm^−1^ is attributed to the carbonyl group, in which a previous study suggested the presence of an ester group in suberin [26]. Faix [27] suggested that the peak is attributed to C=O in unconjugated ketones, carbonyls, and ester groups (commonly of carbohydrate origin). Another author relates the absorption band to the occurrence of the acetyl and uronic ester groups of hemicellulose as well as ρ–coumaric acids in lignin and/or hemicellulose [28]. The bands at 1508 cm^−1^ and 1424 cm^−1^ are assigned to aromatic skeletal vibrations in lignin [27]. The absence of lignin-associated bands is obvious in the FTIR spectra after raw Macaranga was processed into pulp. The disappearance of a distinct peak at 1738 cm^−1^ in all the corresponding spectra indicates the removal of acetyl and uronic ester groups of hemicellulose or the ester linkage of lignin. Furthermore, the FTIR spectra shown in samples of unbleached, bleached, NFC, and NCC display similar fingerprints ascribed to typical cellulose. The broad absorption band at approximately 3338–3378 cm^−1^ is due to the stretching vibrations of hydroxyl groups at the cellulose backbone [29]. The band at approximately 2901–2921 cm^−1^ present in all the processed pulp and nanocellulose is ascribed to the C-H stretching vibration in cellulose. The peak at 1639–1648 cm^−1^ is attributed to the adsorbed water.

**Figure 5:**
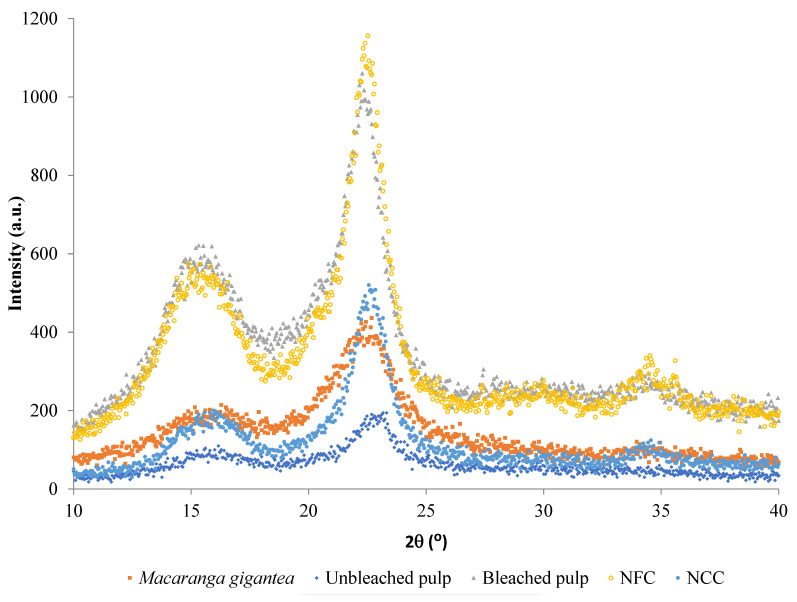
XRD graphs from every processing stage. raw material Macaranga to nanocellulose.

XRD is one of the essential characterization techniques for nanocrystals due to its capability to expose the crystalline order of the material. In addition, it has become one of the basic methods for crystallography studies because of the direct correlation between the diffraction pattern and crystal structure. XRD was conducted on all samples to observe their respective crystallinity changes. The XRD diffraction pattern for all samples corresponds to the cellulose I_β_ polymorph, in which distinctive peaks appear at 2θ values of 15, 16, 22.5, and 34 ascribed to crystal planes 110, 110, 200, and 040, respectively (Figure 5). Cellulose I_β_ is commonly present in the plant kingdom. The estimated crystallinity value was found to increase after the sample was subjected to each treatment. The crystallinity index values were 54%, 55%, 60%, 70%, and 69% for raw Macaranga, unbleached pulp, bleached pulp, NFC, and NCC respectively. The increased value of crystallinity index observed from raw material to nanocellulose can be ascribed to the removal of amorphous fractions such as lignin and hemicellulose.

**Figure 6:**
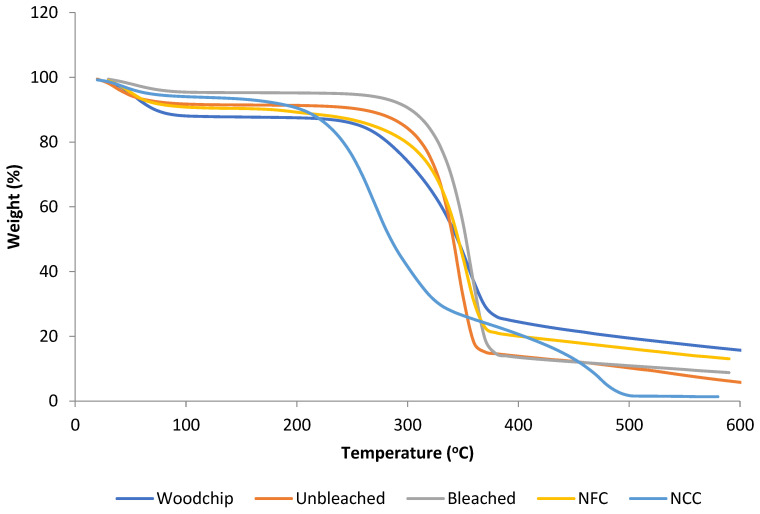
TGA graphs from every processing stage i.e., raw material Macaranga to nanocellulose.

The thermal behavior properties of all samples were evaluated via thermogravimetric analysis. Looking at the thermogram for all the samples, they displayed the same shape except for NCC (Figure 6). It was observed that the wood chips seemed to decompose faster in the initial stage than the rest of the samples. The initial weight loss between 40 and 100 °C is due to water evaporation and moisture removal in the sample material [30]. The second phase of decomposition starts at approximately 200 °C and continues until approximately 380 °C, which is ascribed to the decomposition of hemicellulose and cellulose. Cellulose decomposition generally starts at 270 °C for all the samples except NCC. In the case of NCC, cellulose decomposition starts at 200 °C, followed by a second decomposition between 310 and 490 °C. The lower thermal stability of NCC compared to the rest of the samples is due to the presence of sulfate groups on the surface of NCC, which makes the decomposition much faster. If protonated, the sulfate groups are protic acids that can catalyze degradation, whereas if unprotonated, they undergo elimination reactions that can also lead to a reduction in thermal stability [31].

Moisture is an important element in packaging, as it affects the product quality inside the package, especially for food products. The material used for food packaging must have good barrier properties to ensure that the food is protected from being spoiled. This means that permeability data for the film must be reliable and within the accepted value. In this study, thin films were produced from pure NCC and NFC via the casting method, and they were then evaluated for their permeability properties. Table 4 shows the tensile strength, transparency, air permeance, WVTR, and WVP values for NCC and NFC. Both thin films were observed to have a comparable tensile strength. On the other hand, the NCC thin film was less transparent than the NFC thin film.

Based on the air permeance results, NCC seems to be less impermeable than NFC by almost five-fold. Air permeance indicates the material’s pore structure, which from the findings implies the less porous structure of NFC thin film compared to NCC. In addition, slower air passage is preferred in packaging to avoid microbial contact, particularly in food applications. It is interesting to note that the WVTR value for NCC is more than two times the NFC value. This shows that the NCC thin film absorbs more water vapor than NFC. The WVTR value for NFC is close to the value of the polyester film, whereas NCC is comparable to the oriented polypropylene value [32]. We also noticed interesting expected behavior during thin film preparation, in which the NCC film tends to be more brittle and easily cracked if not handled properly, whereas the film from NFC is more like a plastic material. This is due to the structure network of each film, in which NFC has a compact and dense structure organization that could reduce the penetration of water vapor into the film, whereas NCC is more porous, rigid, and less complicated. Such rigidity and porosity make NCC more brittle than NFC. The air permeance, WVTR, and WVTP properties shown in Table 4 indicate that NFC could be a preferable biobased material for thin film production for packaging applications.

## 4. Conclusions

In conclusion, *Macaranga gigantea* was successfully converted to NCC and NFC by employing a sulfuric acid hydrolysis method and a combination of enzymatic pretreatment and ultrafine grinding methods, respectively. The corresponding nanocellulose had widths below 100 nm, in which NCC and NFC had average values of 6.38 ± 3.92 nm and 13.17 ± 12.71 nm, respectively, thus verifying the conversion and production of nanomaterials from pioneer wood species of Macaranga. FTIR and TGA analyses also revealed the removal of non-cellulosic materials to support the successful conversion of nanocellulose. The value of crystallinity also increased during nanocellulose preparation (from 54% to 70%) which can be contributed to the removal of amorphous fractions. The thin film produced from both nanocelluloses had a WVTR value similar to the value of a thin film made from polyester and oriented polypropylene, indicating its initial potential for sustainable packaging applications. Nevertheless, further in-depth study needs to be performed to evaluate their comprehensive barrier properties for specific packaging requirements.

## Figures and Tables

**Table 1:** Chemical compositions of *Macaranga gigantea*.

Composition	(%)
**Holocellulose**	76.6 ± 0.95
**Alpha cellulose**	40.5 ± 0.43
**Lignin**	33.3 ± 0.8
**Alkali**	9.9 ± 0.3
**Hot water**	2.3 ± 0.2
**Pentosan**	7.8 ± 0.0
**Ash**	1.3 ± 0.1
**Extractives**	0.8 ± 0.2

**Table 2:** Fiber morphology of *Macaranga gigantea* after pulping and bleaching.

Fiber Type	Length (mm)	Width (µm)	Lumen (µm)	Wall Thickness (µm)	Runkel Ratio	Coefficient of Suppleness	Felting Power
**Macaranga raw**	1.23	21.61	16.24	2.69	0.25	75.2	73.2
**Unbleached pulp**	1.17	20.52	15.58	2.47	0.24	75.9	57.0
**Bleached pulp**	1.09	16.0	9.37	3.31	0.41	58.6	56.9

**Table 3:** Width and length of nanocellulose.

Type of Nanocellulose	Width (nm)	Length (nm)
**NCC**	6.38 ± 3.92	392.60 ± 32.09
**NFC**	13.17 ± 12.71	n.d

n.d: not determined.

**Table 4:** Tensile strength, transparency, air permeance, WVTR and WVP values for NCC and NFC.

Material	Tensile Strength (kN/m)	Transmittance (%)	Air Permeance (µm/Pa·s)	WVTR (g/day·m^2^)	WVP (g·µm/m^2^·day·mmHg)
**NCC**	0.64	6	1.24	12.14	9.84 × 10^−2^
**NFC**	0.56	34	0.25	4.58	3.15 × 10^−2^

## Data Availability

Not available.
